# Low response in eliciting neuraminidase inhibition activity of sera among recipients of a split, monovalent pandemic influenza vaccine during the 2009 pandemic

**DOI:** 10.1371/journal.pone.0233001

**Published:** 2020-05-13

**Authors:** Hiroko Ito, Hidekazu Nishimura, Tomoko Kisu, Haruhisa Hagiwara, Oshi Watanabe, Francois Marie Ngako Kadji, Ko Sato, Suguru Omiya, Emi Takashita, Eri Nobusawa

**Affiliations:** 1 Clinical Research Division, Virus Research Center, National Hospital Organization Sendai Medical Center, Sendai, Japan; 2 Hagiwara Clinic, Tokyo, Japan; 3 Influenza Virus Research Center, National Institute of Infectious Diseases, Tokyo, Japan; Sun Yat-Sen University, CHINA

## Abstract

Antibodies against influenza virus neuraminidase (NA) protein prevent releasing of the virus from host cells and spreading of infection foci and are considered the ‘second line of defence’ against influenza. Haemagglutinin inhibition antibody-low responders (HI-LRs) are present among influenza split vaccine recipients. The NA inhibition (NAI) antibody response in vaccinees is worth exploring, especially those in the HI-LRs population. We collected pre- and post-vaccination sera from 61 recipients of an inactivated, monovalent, split vaccine against A/H1N1pdm09 and acute and convalescent sera from 49 unvaccinated patients naturally infected with the A/H1N1pdm09 virus during the 2009 influenza pandemic. All samples were subjected to haemagglutinin inhibition (HI), NAI and neutralisation assays. Most paired sera from naturally infected patients exhibited marked elevation in the NAI activity, and seroconversion rates (SCR) among HI-LRs and HI-responders (HI-Rs) were 60% and 87%, respectively; however, those from vaccinees displayed low increase in the NAI activity, and the SCR among HI-LRs and HI-Rs were 0% and 12%, respectively. In both HI-LRs and HI-Rs, vaccination with the inactivated, monovalent, split vaccine failed to elicit the NAI activity efficiently in the sera of the naive population, compared with the natural infection. Hence, the improvement of influenza vaccines is warranted to elicit not only HI but also NAI antibodies.

## Introduction

Influenza viruses contain two glycoproteins—haemagglutinin (HA) and neuraminidase (NA)—on their surface. The viral infection cycle begins with binding of HA to receptors, sialic acids at the terminal ends of glycoconjugates on the host cell surface, and ends by releasing progeny viruses from the cell surface after the replication in host cells “[1]”. NA works in the final stage of the cycle and cuts off sialic acid from the sugar chain, which is attached in the rough endoplasmic reticulum and matured in Golgi apparatus on the nascent HA on progeny viruses, and the cell-surface glycoconjugates “[1]”. NA dysfunction causes the clustering of progenies on the cell surface “[2]”. Another function of NA in virus entry into cells has been suggested”[3,4]”. HA is a primary target of the antibody response, and antibodies against it prevent the viral entry into host cells. Reportedly, antibodies against NA do not prevent infection itself but inhibit the enlarging infection foci *in vitro* “[2,5]”. *In vivo*, the anti-NA antibody modified the disease and conferred the resistance or protection to infected individuals during the 1968 pandemic of A/H3N2 and to mice and human volunteers in some challenge experiments “[6–8]”. In the 1970s, Kilbourne et al. advocated the concepts of ‘second-line defence’ and the ‘infection-permissive’ immunisation with the NA antigen “[5]”. Some studies have investigated the anti-NA antibody induction by inactivated influenza vaccine “[8–18]”. The inactivated split influenza vaccine contains HA and NA antigens, which are dissociated from the virions by ether treatment “[19]”. In Japan, inactivated, monovalent, split vaccines against influenza A/H1N1pdm09 that emerged in 2009 were approved because their field trials attained 78.1% seroprotection rate (SPR) and 75.0% seroconversion rate (SCR) in the adult population who received the vaccine once “[20]”, and the vaccines cleared the European Medicines Agency’s criteria for effective influenza vaccines “[21]”. However, 78.1% SPR implied that 78.1% of recipients acquired the protection-level immunity, while the remaining 21.9% did not; it is essential determining for the latter population whether they benefit from the vaccination by acquiring some defence mechanisms other than haemagglutinin inhibition (HI) antibody, or not. Hence, we focused on the NA inhibition (NAI) activity in the sera of such a population.

To investigate the antibody response to a virus in a straightforward manner, subjects were preferred to be immunologically naive to the antigen, without pre-existing specific immunity. Nevertheless, previous studies analysed adults who were infected with seasonal influenza viruses “[9–11,15,17,18]”. For collecting naive adult population, the 1968 pandemic of A/H3N2 was a good opportunity “[6–8]”, and the 2009 pandemic of A/H1N1pdm09 also fitted the bill; these pandemic viruses possessed novel HA and novel HA + NA, respectively, which were antigenically markedly different from seasonal viruses “[22,23]”.

An antibody analysis of the serum could help evaluate immunity induced by natural infections or vaccination. The HI test and the neutralisation (NT) test have been extensively used because their titers correlated well with the protection levels of individuals from the infection “[5]”. This study aims to perform the HI, NT and NAI assays on the paired sera from recipients of an inactivated, monovalent, split vaccine against A/H1N1pdm09 and those from unvaccinated patients naturally infected with the A/H1N1pdm09 virus.

For NAI assay, we used A/H1N1pdm09 whole virion as the antigen, instead of using reassortant H6NX or H7NX virus, virus-like particles, or soluble NA from detergent-treated virion, with which pure antibody reaction to the NA molecule would be expected. We aimed to measur the total serum NAI activity in the serum against the natural virus, which may reflect the natural reaction to the virus *in vivo*. It would involve some effects by the anti-HA antibody like its steric hindrance to the anti-NA antibody “[24]” and possible indirect inhibition against the NA’s enzymatic activity through the inhibition of HA activity that is important for the NA activity “[25]”.

## Materials and methods

### Viral antigens for antibody titration

For the HI test, we purchased the HA antigen of A/California/7/2009(H1N1)pdm09, the vaccine strain used for the 2009 influenza pandemic, from Denka Seiken (Niigata, Japan). A/Narita/1/2009(H1N1)pdm09, isolated in Japan and antigenically close to the A/California/7/2009 strain “[26]”, was propagated in Madin–Darby canine kidney (MDCK) cells (Riken BRC Cell Bank, Tsukuba, Japan) and used for NT and NAI tests.

### Vaccination

In this study, we used the monovalent type A influenza vaccine prepared from the egg-grown A/California/7/2009 virus (Kaketsuken, Kumamoto, Japan) as an inactivated split vaccine without an adjuvant. Each vaccinee received a 0.5-mL vaccine containing 15 μg of HA subcutaneously, with only one shot.

### Serum samples

In this study, we primarily sourced the sera from a serum library of the Sendai Medical Center (Sendai, Japan), which stocks the sera of its workers collected annually for the institutional medical check. The sera from 419 hospital workers [age: 18–59 (average ± SD: 34.2 ± 11) years; male:female, 43:376] were collected in June and December (about 4 weeks after the vaccination in 2009). In addition, the sera from 49 unvaccinated patients [age: 4–55 (26.8 ± 12.3)] infected with the A/H1N1pdm09 virus were collected in a clinic (Hagiwara Clinic, Tokyo) at the acute and convalescent phases of 4 weeks–2 months after the illness onset. The infection was confirmed by the isolation of the virus from nasal swab specimens in MDCK cells, followed by HI tests of the culture supernatant for subtyping. All isolated viruses were confirmed to have similar antigenicity with the vaccine strain through the HI test using the standard serum obtained from the National Institute of Infectious Diseases (Tokyo, Japan) for the viral strain surveillance.

In addition, the sera used as negative controls in the NAI test were pooled sera collected from 10 children (age: <1 year) in 2006; all their HI antibody titers were <1:10 to the A/H1N1pdm09 virus.

### HI and NT assays

We performed the HI assay using the method recommended by the World Health Organization (WHO) “[27]”. The serum samples were treated with the receptor-destroying enzyme II (Denkaseiken, Niigata, Japan) for destroying non-specific inhibitors, followed by removing the xenogeneic agglutination factor against GRBC(Guinea pig red blood cells). Furthermore, the NT assay was conducted using the microneutralisation method recommended by the WHO “[27]”.

### NA and NAI assays

We performed the NA and NAI assays based on the modified Warren’s thiobarbituric acid (TBA) method “[9–11]”, per the WHO recommendation “[28]”. Our assays are almost similar to the miniaturised TBA methods by Sandbulte et al., who used small amounts of reagents and microplate wells “[29]” instead of glass tubes, except that we doubled the reaction volume. Then, the supernatant of the butanol phase in the final reaction mixture containing sialic acid, which was released by NA from the substrate fetuin and chemically coloured, was transferred to a flat-bottomed 384-well microplate; the optical density (OD) was measured at 550 nm using a spectrometer (Thermo Scientific Multiskan FC, MA, USA). The NA activity was defined as the value obtained following the subtraction of the OD value of the control from that of the sample, and the NAI assay was conducted as follows. The antigen was adjusted to such that its NA titre was OD 0.45–0.85. Both test and control sera were serially diluted and reacted with the antigen in the microplate well, followed by a further reaction with the substrate fetuin and the released sialic acid was measured as the OD value. The serum dilution that corresponds to a value 50% in the following equation is defined as the NAI_50_ titre of the tested serum: OD value of tested serum/OD value of control serum × 100 (%) “[28]”. The NAI titre was defined as the dilution of the test serum at the initial point of the titration, which finally provided the NAI_50_. In this study, all assays were performed, at least, in duplicate. The results of our method correlated well with those by the original tube method recommended by the WHO (by Spearman’s analysis, *R*^2^ = 0.74, *r*_s_ = 0.81483, *P* < 0.01).

### Statistical analyses

All statistical analyses were performed using SPSS software version 25.0 (IBM, NY). Discreet data for the number of paired sera in which seroconversion occurred by the vaccination or natural infection, of HI-low responders (HI-LRs) and HI-responders (HI-Rs) were analysed to assess the degree of significance in differences by Fisher’s exact tests, respectively. Using the Spearman’s rank correlation, we analysed the correlation among titers in HI, NAI and NT activities of the sera.

### Ethical statement

This study was approved by the Ethical Committee of the Sendai Medical Center, National Hospital Organization (Sendai, Japan), and we obtained written informed consent from all donors of sera used in this study or their guardians.

## Results

**Fig 1:**
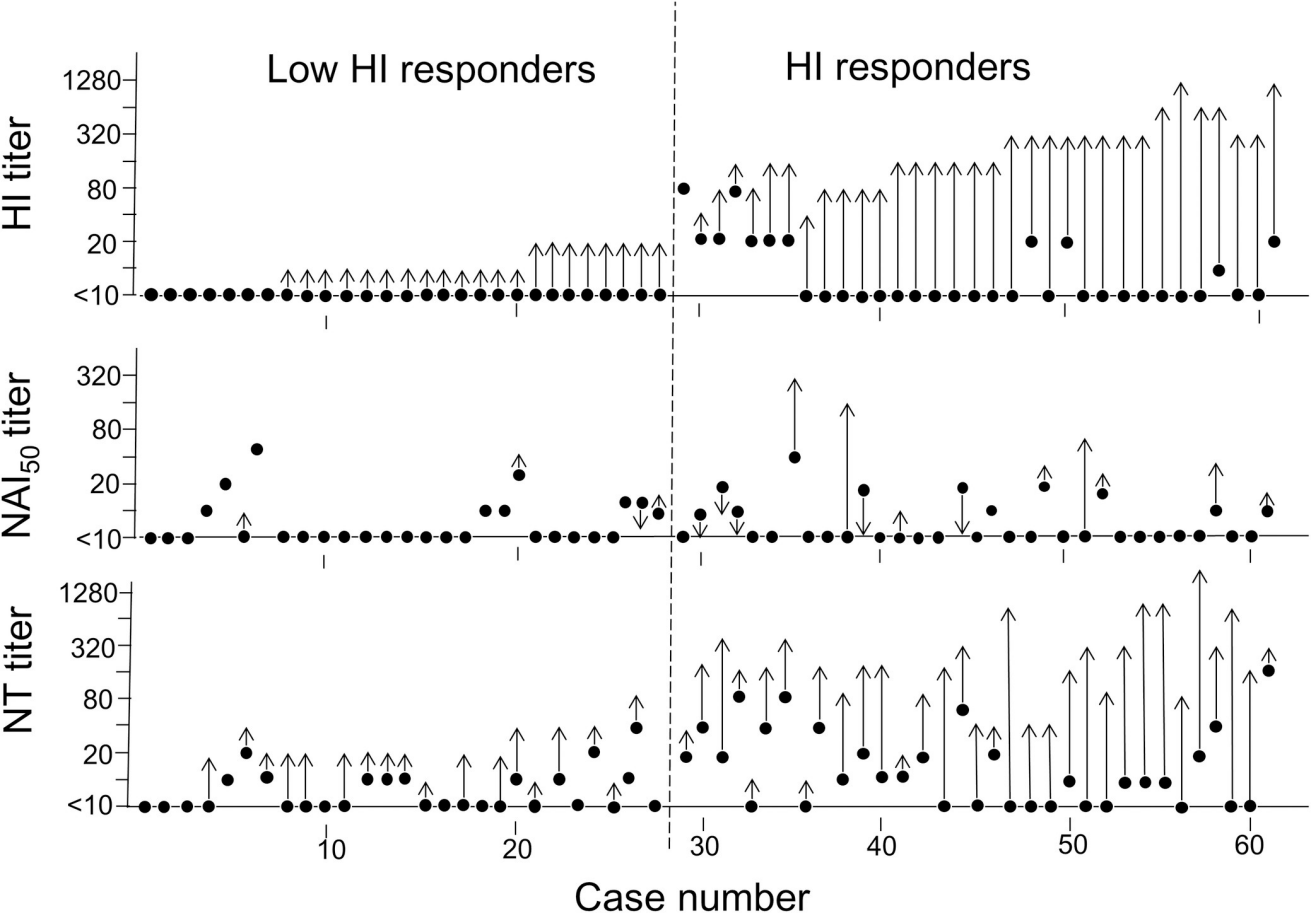
A shift of haemagglutinin inhibition, neuraminidase inhibition and neutralisation titers in individual paired sera of 61 vaccinees. Vaccinees’ case number (abscissa) and the titers of the sera (ordinate). Solid circles, pre-vaccination titers; arrowheads, post-vaccination titers. Only circles were cases without a shift between the paired sera. The dotted line separates the haemagglutination-inhibition in low responders (HI-LR) and responders (HI-R) groups.

**Fig 2:**
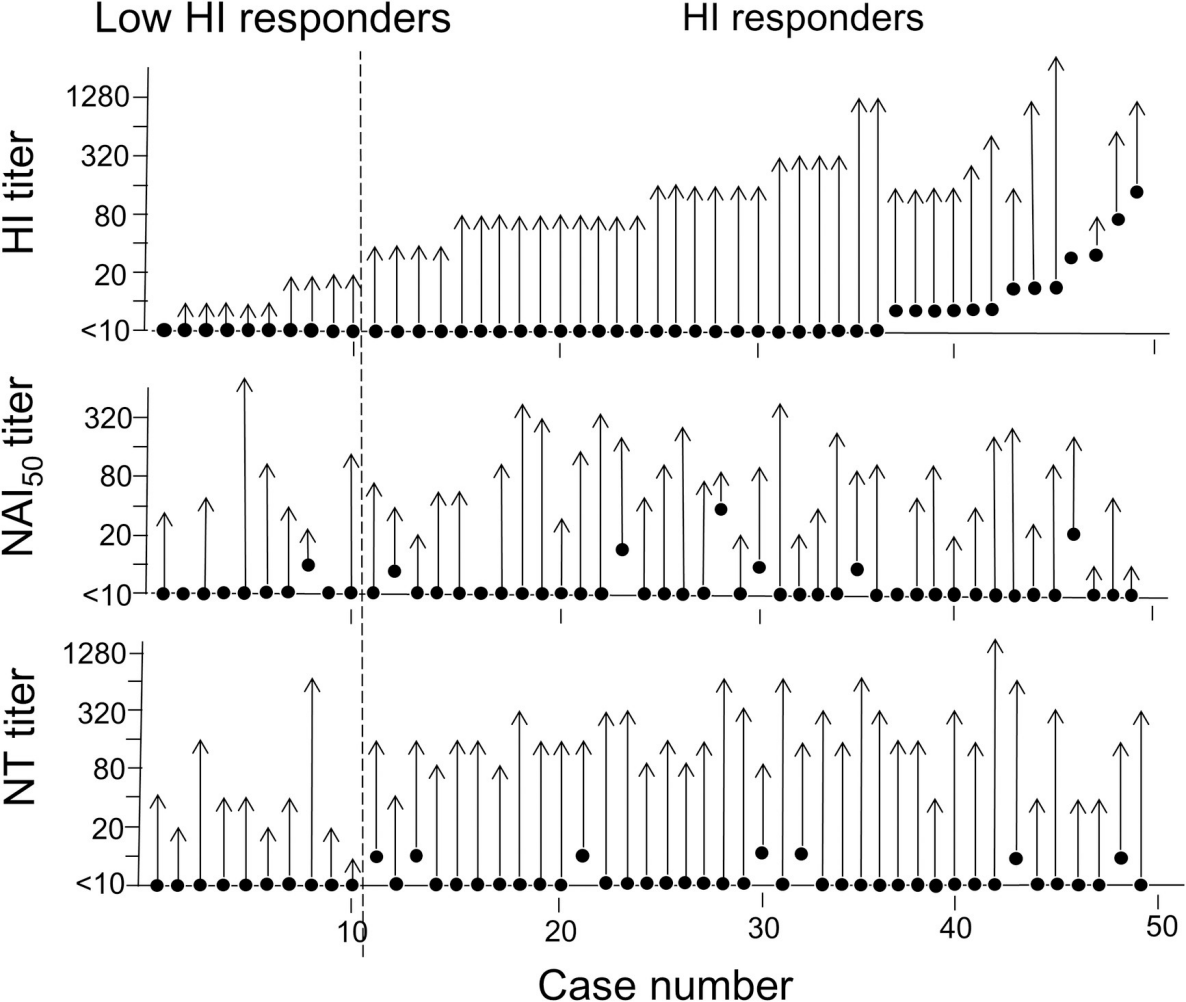
A shift of haemagglutinin inhibition, neuraminidase inhibition and neutralisation activities in individual paired sera of 49 naturally infected cases. Case number (abscissa) and the activities of the sera (ordinate). Solid circles, acute-phase titers; arrowheads, convalescent-phase titers. Only circles were cases without a shift between paired sera. The dotted line separates the haemagglutination-inhibition in low responders (HI-LR) and responders (HI-R) groups.

Influenza A/H1N1pdm09 viruses circulated in Japan in 2009 and were antigenically similar to the A/California/7/2009(H1N1)pdm09 vaccine strain “[26]”. In this study, we investigated HI titers against this strain of 419 pre- and post-vaccination sera and 49 acute and convalescent sera from unvaccinated patients infected with the A/H1N1pdm09 virus. Consequently, the SPR increased from 5% to 76% of vaccinees, suggesting that 99 of 419 vaccinees (24%) did not attain >1:40 after the vaccination, which was considered the minimum titre required for protection “[21]”; we defined such individuals as HI-LRs and those who attained the protection level as HI-Rs. We randomly selected 61 cases of vaccinees (28 and 33 cases from HI-LR and HI-R groups, respectively) and analysed the NT and NAI activities of their paired sera. Furthermore, we analysed the paired sera from natural infection of 49 cases similarly. “Figs 1 and 2” show the titers in each case. “Tables 1 and 2” summarise the statistical analyses. In this study, we found the following. (1) Irrespective of HI-LR or HI-R, an increase in the NAI titer was rare in vaccinees; the SCR was 0% in 28 HI-LRs, with only 4 (12%) of 33 HI-Rs “Fig 1; Table 1”. (2) However, a significant NAI titer increment was recognised in naturally infected cases “Fig 2; Table 1”: the SCR was 60% in HI-LRs [geometric mean titer (GMT) elevation from 5 to 34] and 87% in HI-Rs (GMT increase from 6 to 72), (3) The SCRs in NAI and NT were markedly low in vaccinees compared with naturally infected patients both in HI-LR and HI-R groups “Table 1”. Age ranges of the four groups were—median:38.5, 25.0, 24.0 and 25.0 years in vaccinee HI-LRs, vaccinee HI-Rs, infected HI-LRs and infected HI-Rs, respectively (by ANOVA test, *P* < 0.001). (4) We observed statistically significant correlations between HI and NT in HI-R in both vaccinees and naturally infected groups (*P* = 0.004 and 0.002, respectively), but the correlation was not recognised between NAI and NT titers even in the latter group, as well as between NAI and HI titers “Table 2”. However, the analysis of the data of individual cases revealed that among seven infected cases with increased NAI titer (from <10 to about 40–640) of 10 HI-LRs, six cases correlated to those with 4- to 256-fold increase in the NT titers. Suggesting that the NAI activity possibly contributed to the NT activity “Fig 2”.

**Table 1:** Comparisons of serum reactions between vaccine and natural infection groups.

			vaccinees	Naturally infected	p
Low HI responders	HI	SCR (%)	0	0	NA
		GMT	5→10	5→12	
	NAI	SCR (%)	0	60	0.002
		GMT	7→8	5→34	
	NT	SCR (%)	7	60	0.002
		GMT	7→14	5→43	
HI responders	HI	SCR (%)	91	95	0.235
		GMT	9→201	8→172	
	NAI	SCR (%)	12	87	<0.001
		GMT	7→9	6→72	
	NT	SCR (%)	79	100	0.003
		GMT	14→144	6→178	

NA: Not applicable

**Table 2:** Correlations among three activities of sera of vaccine and natural infection groups.

		Vaccinees				Naturally infected			
		NAI		NT		NAI		NT	
Low HI responders	HI	-0.056	(p = 0.338)	0.121	(p = 0.270)	-0.041	(p = 0.456)	-0.137	(p = 0.353)
	NAI	-	-	0.077	(p = 0.348)	-	-	-0.064	(p = 0.430)
HI responders	HI	0.113	(p = 0.266)	0.450	(p = 0.004)	-0.015	(p = 0.465)	0.449	(p = 0.002)
	NAI	-	-	-0.009	(p = 0.481)	-	-	0.223	(p = 0.087)
Total	HI	-0.017	(p = 0.450)	0.782	(p<0.001)	0.154	(p = 0.146)	0.588	(p<0.001)
	NAI	-	-	0.009	(p = 0.474)	-	-	0.229	(p = 0.057)

## Discussion

Two major ways for assaying the NAI activity are the modified Warren’s TBA method “[9–11]”, which has been recommended by the WHO for a long time, and the ELLA “[30–32]”. Both use a highly sialylated glycoprotein, the fetuin, as a substrate; while the former chemically measures sialic acid released by NA, the latter detects the desialylated fetuin on the microplate with the labelled lectin. The TBA method requires manipulation of large volumes of toxic arsenic acid and could not titrate a large number of samples at once; Sandbulte et al. enabled it by miniaturising the reactions in microplate wells “[29]”, consistent with our method. Our findings on the SCR of the vaccination were very low in HI-LRs and HI-Rs (0% and 12%, respectively), but the reliability of our NAI assay system was supported, at least, by the results of the high SCR in the natural infection group using the same assay system.

To ascertain whether our findings were inferior to those of other studies, we reviewed previous studies “S1 Table”. We collected studies that defined the antibody-rise as >4-fold. Only our study used the A/H1N1pdm09 strain as the antigen for vaccination and NAI titration; however, it could not be attributable to low reactions. A study performed similar to this study, except that the seasonal vaccine was used, exhibited fundamentally similar results “[29]”. For the antigen content of vaccines, the HA contents expressed in μg units are presently substituted for the whole-virion antigen contents, and two studies conducted in 1970s used other antigen units, i.e. chicken cell agglutination (CCA) unit and haemagglutination (HA) unit “[9,10]”. The results of vaccinations and those of different definitions of vaccine contents should be non-comparable.

A difference in assaying, TBA method or ELLA, did not seem to affect the results markedly “[11,18]”. In addition, differences in vaccination pathways—subcutaneous or intramuscular—and differences in the types of virion antigens used for NAI assay—relevant viruses or recombinant viruses possessing irrelevant HA “[29]”—seemed not to affect the results. Reportedly, the natural infection readily activated NA-reactive B cells but immunisation with the current influenza vaccine could not “[33]”.

The augmentation of anti-NA antibody, as well as anti-HA antibody, is a desirable ability for influenza vaccines. If we can elucidate the exact reason for the low efficacy in the split vaccine of inducing NAI antibody in the naive population, it could result in the development of a more potential vaccine.

The low NAI inducibility could be speculated to some loss of immunogenicity or the amount of the NA contained in the vaccine or both in any process in manufacturing the vaccine. Using immunochromatography, Tanimoto et al. estimated that the NA content in Japanese seasonal split-product vaccines was approximately 2.1–5.3 μg/dose, a relatively low amount compared with those of HA (15–25 μg/dose) “[34]”. Using mass spectrometric analyses, Creskey et al. reported that NA contents of monovalent pandemic vaccines in 2009 varied among products, and their data revealed that the ratios of NA contents to HA content ranged from one-third to one-tenth “[35]”. Incidentally, a report of a vaccination study by Kilbourne et al. is interesting in that the SCR was enhanced as the purified NA content increased “[11]”. In addition, Cate et al. reported that a four-fold SCR increase in the anti-NA antibody was attained even in the elderly population when the vaccine content was increased four-fold from 15 to 60 μg/dose on an HA basis “[15]”. Reportedly, the conventional vaccine supplemented with purified NA molecules protected mice more efficiently from the influenza virus infection “[36]”. Hence, it would also be interesting to ascertain whether vaccines supplemented with some adjuvants can elicit the anti-NA antibody more effectively.

## Conclusions

During the 2009 influenza pandemic, vaccination of the inactivated, monovalent, split vaccine failed to efficiently elicit the NAI activity in the sera of the naive population, compared with the natural infection, especially in HI-LRs. Hence, some methodology for influenza vaccines that efficiently induce not only HI but also NAI antibodies should be explored to provide vaccinees with the ‘second-line defence’ against influenza virus infections.

## Supporting information

S1 Table This is the summaries of study designs and results among reports on NAI reactions in recipients of inactivated influenza vaccine. (TIF)
