# Supplementary figures and images for: Low response in eliciting neuraminidase inhibition activity of sera among recipients of a split, monovalent pandemic influenza vaccine during the 2009 pandemic
Source: PLoS One. 2020 May 13;15(5):e0233001. doi: 10.1371/journal.pone.0233001 (PMC7219752; doi:10.1371/journal.pone.0233001)

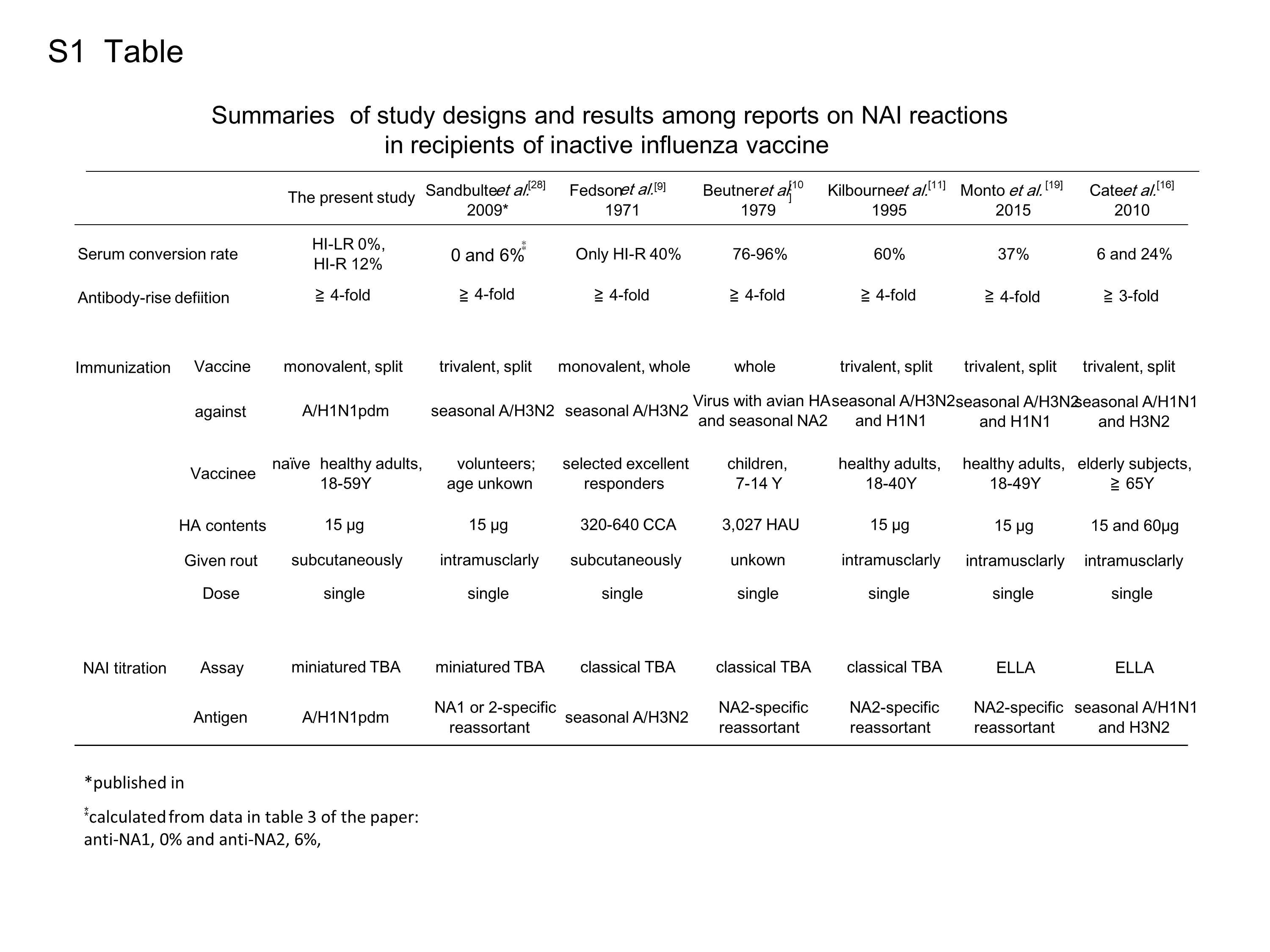

Supplement: S1 Table — (TIF) [file pone.0233001.s001.TIF]
